# Review of Current Strategies for Delivering Alzheimer’s Disease Drugs across the Blood-Brain Barrier

**DOI:** 10.3390/ijms20020381

**Published:** 2019-01-17

**Authors:** Ka Hong Wong, Muhammad Kashif Riaz, Yuning Xie, Xue Zhang, Qiang Liu, Huoji Chen, Zhaoxiang Bian, Xiaoyu Chen, Aiping Lu, Zhijun Yang

**Affiliations:** 1 School of Chinese Medicine, Hong Kong Baptist University, Hong Kong, China; 16483081@life.hkbu.edu.hk (K.H.W.); kashif@life.hkbu.edu.hk (M.K.R.); 17433606@life.hkbu.edu.hk (Y.X.); bzxiang@hkbu.edu.hk (Z.B.); cxyu2016@hkbu.edu.hk (X.C.); 2 The Key Laboratory of Syndrome Differentiation and Treatment of Gastric Cancer of the State Administration of Traditional Chinese Medicine, Yangzhou 225001, China; zhangxueflora@163.com; 3 Institute of Translational Medicine, Medical College, Yangzhou University, Yangzhou 225001, China; 4 School of Traditional Chinese Medicine, Southern Medical University, Guangzhou 510515, China; gzlq2002@163.com (Q.L.); chenhuoji2005@126.com (H.C.); 5 Changshu Research Institute, Hong Kong Baptist University, Changshu Economic and Technological Development (CETD) Zone, Changshu 215500, China

**Keywords:** brain delivery, blood-brain barrier, central nervous system, nanomaterials, Alzheimer’s disease, cell-penetrating peptide

## Abstract

Effective therapy for Alzheimer’s disease is a major challenge in the pharmaceutical sciences. There are six FDA approved drugs (e.g., donepezil, memantine) that show some effectiveness; however, they only relieve symptoms. Two factors hamper research. First, the cause of Alzheimer’s disease is not fully understood. Second, the blood-brain barrier restricts drug efficacy. This review summarized current knowledge relevant to both of these factors. First, we reviewed the pathophysiology of Alzheimer’s disease. Next, we reviewed the structural and biological properties of the blood-brain barrier. We then described the most promising drug delivery systems that have been developed in recent years; these include polymeric nanoparticles, liposomes, metallic nanoparticles and cyclodextrins. Overall, we aim to provide ideas and clues to design effective drug delivery systems for penetrating the blood-brain barrier to treat Alzheimer’s disease.

## 1. Introduction

Alzheimer’s disease (AD) is a chronic neurodegenerative disorder associated with accumulation of amyloid beta and intracellular neurofibrillary tangles in the brain [1]. It is estimated that 5.7 million people in the US have AD, of whom 5.5 million are aged 65 years or older. AD is the sixth leading cause of death in the US. The disease kills more than the combined mortalities of breast cancer and prostate cancer. The mortality rate has increased 89% since 2010 [2]. AD therapy can be divided into non-medical and medical. Non-medical treatment mainly aims to improve the quality of life or maintain the cognitive and daily activity abilities of patients. So far, there are six FDA approved prescription drugs to treat AD. However, these drugs can only relieve symptoms of the disease temporarily and none one of them has proven the ability to cure or stop the progression of the disease [2,3]. Furthermore, the efficiency of the drugs varies from person to person and from stage to stage and the drugs always accompany with side effects such as nausea, diarrhea and vomiting [4]. At the same time, failures in AD drug development happen frequently. In some cases, trials employing small molecules or those using immunotherapies were not able to show significant difference between drug and placebo; some revealed unpredictable toxicity [5]. Although there are clinical trials showed encouraging results, for example, BAN2401 can significantly reduce cognition and remove amyloid from the brain in phase 2 study [6], there is still an urgent need for more treatment approaches. If the situation of treating AD is not improved, the number of patients over 65 years old may rise to 13.8 million by 2050 in the US [2].

In order to achieve successful treatment of AD, the role of the blood-brain barrier (BBB) has to be considered. The BBB is a specialized structural, physiological and biochemical barrier; it serves as the first interface between the changeable environment of blood and the extracellular fluid in the central nervous system (CNS) [7]. The BBB regulates the homeostasis of the nervous system by strictly controlling the movement of small molecules or macromolecules from the blood to the brain. It only permits selective transport of molecules that are essential for brain function. In detail, more than 98% of small molecule drugs and almost 100% of large molecule drugs are precluded from drug delivery to brain [8]. Water-soluble molecules in the blood are prevented from entering the CNS and lipid-soluble molecules are reduced by the function of enzymes or efflux pumps [9]. These properties of the BBB make the CNS one of the most complicated microenvironments of the body and limit the development of novel drugs for CNS diseases. Drug delivery system (DDS) has the potential to be effective in CNS diseases treatment as it shows various advantages when compared to chemotherapy. These advantages include delivering the drug to a specific site, protecting the drug from clearance by the circulatory and immune systems, altering the physicochemical properties of drugs, reducing the dose and controlling the drug release [10,11,12]. They make DDS an attractive option for treating AD.

In this review, different strategies of designing DDS for penetrating the BBB to treat AD have been described and discussed. First, the pathology of AD and biological and physicochemical properties of the BBB have been reviewed as such properties determine the targeting strategies of DDS. In the second part, various DDSs have been examined. Both the merits and drawbacks of the mentioned systems have been summarized. In the last part, suggestions for future development of DDSs towards AD have been proposed. Overall, by presenting and comparing the numerous DDSs now available, we aim to provide ideas and clues for designing systems specifically effective for treating AD.

## 2. Pathophysiology of Alzheimer’s Disease

The cause of AD is still not fully understood. Research suggests that signs associated with AD can be found in the brain 20 or more years before the onset of symptoms [2,13,14]. It may be possible that the initial changes in the brain can be compensated. When the changes are no longer reversible, symptoms gradually become apparent [15]. First, cognitive decline happens and then memory loss will develop. In the most serious cases, basic daily functions are affected. Several hypotheses have been proposed to give an explanation and the most popular are the amyloid hypothesis and the tau hypothesis.

### 2.1. Amyloid Hypothesis

The amyloid hypothesis suggests that the pathogenesis of AD is due to the extracellular accumulation and aggregation of Amyloid β (Aβ) peptides in the brain [16,17]. Aβ is generated because of the cleavage of the trans-membrane amyloid precursor protein (APP). The accretion of Aβ peptides leads to a series of neurotoxic issues [18,19]. It leads to loss of mitochondrial function. By localizing mitochondrial membranes and blocking the transport of proteins to mitochondria, it results in mitochondrial damage [20]. It is also reported that Aβ peptides react with metal ions in the brain to produce reactive oxygen species (ROS) and increase oxidative stress [21]. Aβ peptides can also disrupt calcium homeostasis by changing the concentration of calcium ions. It also triggers unregulated flux of calcium through the plasma membrane [22,23]. At the same time, microglia release inflammatory mediators such as inflammatory cytokines and chemokines to cause neuroinflammation due to the microglia activation by Aβ peptides [24]. The relationship between Aβ peptides and neurotoxic issues is not unidirectional as they trigger each other in a positive feedback loop. Production of Aβ peptides leads to neurotoxic responses, while such responses impede the metabolism of APP and result in accumulation of Aβ peptides [21,22,23,24,25,26].

### 2.2. Tau Protein

According to the amyloid hypothesis, Aβ peptides accumulation initiates hyperphosphorylation of tau protein. Tau protein is a microtubule-associated protein (MAP) that is encoded by the microtubule-associated protein tau (MAPT) gene. It is a highly soluble protein to stabilize microtubules. Tau proteins, which are abundant in neurons of the CNS, exist as six isoforms in brain tissue [25]. In the brain of AD patients, tau is hyperphosphorylated at least three folds (molar ratio) higher than that in the normal brain [25,27]. The abnormal hyperphosphorylation of tau plays a significant role in neurofibrillary degeneration. During hyperphosphorylation, tau proteins misfold and accumulate into paired helical filament (PHF) tau and also neurofibrillary tangles (NFTs) [28]. NFT may reduce normal tau function, compromise normal cellular functions and disrupt tau-mediated regulation of microtubule dynamics, finally resulting in neurodegeneration [29].

### 2.3. Others

According to the genome wide association studies (GWAS), there are at least 20 genes that contribute to the AD onset and evolution [30]. Apolipoprotein E (ApoE) is one of the best well known genes in AD. ApoE isoforms can regulate Aβ aggregation and clearance in the brain and participate in regulating glucose metabolism and neuronal signaling [31]. ApoE can also regulate the integrity of tight junctions and ApoE deficiency can lead to BBB leakage [32]. There are other genes that also affect the BBB physiology and molecule exchanges across the BBB. ABCA7 gene is closely linked to excessive accumulation of amyloid peptides while ABCA7 gene downregulation might affect cholesterol and amyloid exchanges at the BBB [33]. In the following part, more details related to the BBB have been discussed.

## 3. Blood-Brain Barrier

The BBB is an active interface with an average surface area of about 12–18 m^2^ for a human adult [34]. Only a few regions, such as circumventricular organs (CVO), lack the BBB. But it has to be noticed that a complicated system surrounding CVO preventing blood-borne compounds from entering the BBB-protected region [35]. The total length of capillaries in a normal human adult brain is about 400 miles; that length is shorter in the brains of AD patients [36]. Due to the degeneration of the capillaries, transportation of nutrients and essential substances across the BBB and the clearance of neurotoxins such as Aβ peptides from the brain are reduced [36,37,38]. The BBB is a capillary wall mainly formed by brain endothelial cells and its basement membrane, with the presence of cell-cell junctions to keep the integrity of the brain microvasculature. Cell-cell adhesion can be categorized into adherens junctions and tight junctions. The tight junctions preclude the paracellular transport of movement of most of the molecules and ions. Therefore, the transport of most of the molecules between the vascular system and brain is mainly through the transcellular transport [39,40]. There are also components that take part in the formation and functions of the BBB. The brain capillaries are surrounded by the end-feet of astrocytes, which are involved in the regulation of ion concentration and clearance of neurotransmitters [41]. Pericytes are vascular mural cells locating on the abluminal aspect of endothelial cells and astrocyte end-feet wraps around the pericytes layer, which are able to regulate the blood flow by controlling capillary diameter. Pericytes are important for BBB formation and for downregulation of transcytosis activity when endothelial cells mature. Therefore, pericytes are also related to the transportation across the BBB [42]. Other cellular components such as neurons and microglia also take part in forming the BBB. The functional interactions and signaling between the above-mentioned neurons and non-neuronal cells form a dynamic functional unit, which is called the neurovascular unit [43]. All the components are essential for normal functions, stability of the BBB and the response of the BBB to pathophysiological stimuli. The dilemma for the treatment of AD and other brain diseases is not only due to the lack of effective therapeutic molecules but also due to their inability to penetrate the basement membrane of the BBB and reach the specific target site to treat the disease [44]. Many factors influence the entry of candidate drugs into the brain parenchyma. In next section, we have discussed transportation of molecules across the BBB.

## 4. BBB Permeation Mechanisms

**Figure 1:**
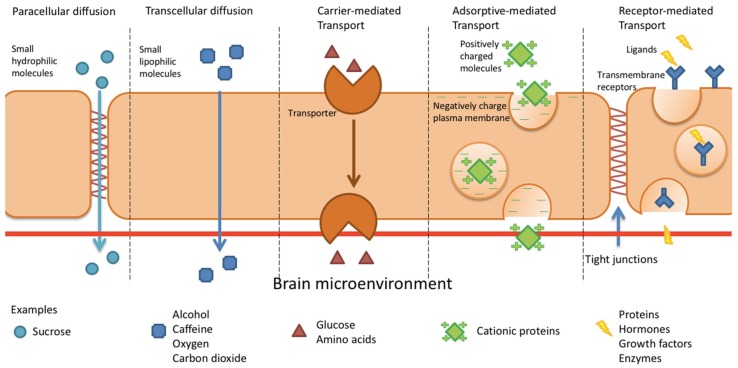
Pathways for BBB penetration. Adapted with permission from Elsevier, *Mol. Med. Today*, Abbott, N. J. & Romero, I. A., Transporting therapeutics across the blood–brain barrier, 1996 and permission from Springer Nature, *Nat. Rev. Neurosci.*, Abbott, N.J. et al. Astrocyte–endothelial interactions at the blood–brain barrier, 2006.

There are several transport routes for molecules to cross the BBB (Figure 1). A few small hydrophilic and lipophilic molecules can enter the brain by paracellular and transcellular diffusion. Other substances, such as amino acids, peptides and nucleosides, may enter the brain through carrier-mediated transport, receptor-mediated transcytosis and/or adsorptive-mediated transcytosis.

### 4.1. Paracellular and Transcellular Diffusion

Passive diffusion is the primary means of most molecules and therapeutic compounds to enter the brain from the bloodstream [45]. On a molecular level, the BBB consists of a highly anisotropic lipid bilayer [46]. Normally, substances that are able to penetrate the BBB by passive diffusion are highly lipid soluble with molecular weights less than 400–500 Da; while molecules with neutral charge in physiological pH range of the brain show stronger permeability. The high polar surface area of the molecule should not be greater than 80 Å^2^ [45,47]. Many molecules entering the brain through passive diffusion are subsequently excluded to prevent entry or removed by efflux pumps once entering the brain through passive diffusion. Small hydrophilic molecules such as sucrose can cross the BBB paracellularly in the direction of the concentration gradient between endothelial cells. Due to the presence of tight junctions, such substances can get into the brain through this pathway to only a limited extent. And it is usually negligible in the consideration of drug discovery. Small lipophilic molecules such as alcohol, caffeine and gaseous molecules such as oxygen and carbon dioxide can diffuse through the cell membrane transcellularly [39,48,49].

### 4.2. Carrier-Mediated Transport

For the molecules such as glucose and amino acids that cannot diffuse through the cell membrane, they can enter the CNS through carrier-mediated influx. There are various specific transporters which may be specific to one or several molecules [39]. Glucose and amino acids can bind to a protein transporter on one side of the membrane. By triggering conformation change of the membrane, the compounds can pass through the membrane in the direction of the concentration gradient [44]. Glucose transporter GLUT1 is important for glucose uptake in the brain. Hypoglycemia can up-regulate the concentration of GLUT1 while hyperglycemia cannot. Therefore, sugar transport the BBB can be related to the cell surface GLUT1 levels [40,50]. From recent findings, GLUT1 is down-regulated in mice overexpressing Aβ. It results in the degeneration of cerebral microvascular, reduction of Aβ clearance and neurodegeneration [51]. The observations described above suggest that GLUT1 can act as a potential therapeutic target for AD treatment.

### 4.3. Adsorptive-Mediated Transcytosis

In adsorptive-mediated transcytosis, positively charged molecules interact with the negatively charged plasma membrane, thereby initiating endocytosis which leads to transcytosis [52]. Protein receptors are not involved in this pathway; thus, the lack of specificity may lead to non-discriminated adsorption of molecules from the blood. The electrostatic binding depends on the affinity of the moieties with binding sites and capacity of the binding sites. Adsorptive-mediated endocytosis usually has higher capacity and lower affinity as compared to receptor-mediated transcytosis [53]. The former pathway is used to transport cationic proteins into the brain. Although the non-specific transcytosis is not ideal for targeted drug delivery, there are still some successful brain delivery strategies based on adsorptive-mediated transcytosis; these usually involve cationic proteins or cell penetrating peptides (CPP) such as Syn-B vectors or TAT peptides.

#### 4.3.1. Syn-B Vectors

CPPs are short amphipathic or cationic peptides with 10 to 27-amino acid residues [54]. Certain CPPs are able to cross cell membranes without the aid of a receptor by binding to the negatively charged cell membrane strongly through non-specific electrostatic interactions. Syn-B vectors are examples; they utilize adsorptive-mediated endocytosis to cross the BBB. Syn-B vectors are peptides derived from a natural mammalian antimicrobial peptide protegrin 1 (PG-1) with high affinity for biological membranes [55]. PG-1 is an 18-amino acid peptide isolated from porcine leukocytes with an antiparallel beta-sheet structure stabilized by two disulfide bridges [55,56]. A variety of liner analogues of PG-1 have been designed by the removal of cysteine residues. These vectors are able to cross the membrane without disrupting it. The mechanism of brain uptake of these vectors does not involve any stereospecific receptor [57]. The syn-B vectors have also been shortened to less than 10 amino acids for optimizing their translocation properties. The families of these peptides have been used for brain delivery of poorly brain-penetrating drugs. For example, l-SynB1, l-SynB3 and d-SynB3 significantly increase the brain uptake of doxorubicin by 30-fold [57]. SynB1 also enhances the uptake of benzylpenicillin (B-Pc) in the brain without compromising the BBB integrity [58].

#### 4.3.2. TAT-Derived Peptides

TAT-derived peptides, another example of CPPs, have also been studied extensively for drug delivery. They are derived from the transcription activating factor of human immunodeficiency virus (HIV-1). TAT protein is an 86-amino acid protein and the minimal functional domain for translocation is the domain from 48–60 [59]. Analogues of the peptides have been synthesized and studied. The cell penetrating ability of TAT is highly related to its arginine residues. Substituted arginine residues in TAT resulted in obvious decrease in the penetrability [60]. Although TAT transduction of molecules does not show specificity, the ability to penetrate the BBB made TAT the most widely used CPP for delivering proteins and nanoparticles to the brain. TAT-conjugated quantum dots have been investigated for labeling brain tissue without manipulating the BBB [61]. For AD treatment, a study reported that by linking TAT peptides with natural variant of Aβ carrying the A2V substitution, the resulting hexamer [Aβ1-6_A2V_TAT(D)] strongly inhibited oligomerization, amyloid fibril formation and Aβ-dependent neurotoxicity in vitro. It also inhibited Aβ aggregation and cerebral amyloid deposition in vivo after short-term treatment. However, there was an increase in amyloid burden, even though [Aβ1-6_A2V_TAT(D)] prevented cognitive deterioration after long-term treatment [62]. It might be due to the intrinsic ability of TAT to induce Aβ deposition, tau phosphorylation and subsequent neuronal death in AD development [63,64]. These issues have to be taken into account when designing systems to penetrate the BBB for AD treatment. Nonetheless, recent studies have shown a novel fusion peptide created by linking the brain-derived neurotrophic factor (BDNF) with TAT can efficiently target multiple molecular pathways in the brain. Due to the large molecular weight, BDNF is not able to cross the BBB. However, linked with TAT, the fusion peptide could efficiently enter the brain and modulate Aβ and tau pathologies in APPswe mice [65]. This strategy combined the advantages of the penetrating capacity of TAT and the selectivity of BDNF. Double functionalization is definitely a strategy worth exploring in the treatment of CNS diseases, including AD.

There are also other peptides that have been reported to have cell-penetrating ability and therapeutic effects against AD. The peptide, R_8_-Aβ_25–35_, created by combining polyarginines (polyR) with Aβ_25–35_, has shown significant efficacy in reducing Aβ accumulation and improving cognitive functions of APP/PS1 double transgenic mice [66]. The highly hydrophilic and cationic nature of polyarginines peptides is responsible for the charge repulsion that makes endocytosis possible. The working principle is similar to that of the above-mentioned TAT-BDNF fusion peptides [65].

### 4.4. Receptor-Mediated Transcytosis

Receptor-mediated transport uses the vesicular trafficking machinery of brain endothelial cells to deliver a range of proteins, hormones, growth factors, enzymes and plasma proteins to the brain [44,67]. During receptor-mediated transcytosis, macromolecular ligands first bind with specific transmembrane receptors, followed by triggering endocytosis. Membrane invagination takes place and the complexes of receptors and ligands are pinched off into a vesicle. Then the vesicle is transported across the endothelial barrier. At the same time, exocytosis happens. The complexes are dissociated, thereby releasing the ligands into the brain. The number of transmembrane receptors is limited; thus, binding and uptake process continues until the capacity of receptors is saturated [68]. Besides adsorptive-mediated transcytosis, receptor-mediated transcytosis is another major route for targeted drug delivery into the BBB. It involves the use of targeting ligands to modify the surface of various DDSs. Many receptors are known to take part in receptor-mediated transcytosis, including: transferrin receptor, lactoferrin receptor, insulin and insulin-like growth factor receptors, leptin receptor, heparin-binding EGF and tumor necrosis factor receptor. In this connection, we have discussed on several receptors that are commonly used for targeted drug delivery in AD therapy.

#### 4.4.1. Transferrin Receptor

Transferrin receptor used as a target for drug delivery to the brain has been studied for a long time. Transferrin receptor is a transmembrane glycoprotein consisting of two subunits which are linked by a disulfide bond and each of the subunits can bind with one transferrin molecule. Transferrin is present in blood plasma and brain extracellular fluids while the transferrin receptor is expressed in many tissues. In the brain, the transferrin receptor can be found in endothelial cells, epithelial cells, neurons and glial cells. Cellular uptake and translocation of transferrin-bound iron is regulated by transferrin receptor via receptor-mediated transcytosis [69]. It is reported that the number of transferrin receptors in the hippocampus of AD patients is less than normal, while in cerebral microvessels it is normal [70]. Thus, the transferrin receptors on the endothelial cells in the brain are relatively abundant and they can be employed as a target in brain delivery for transferrin binding. The applications of transferrin for molecular targeting are always associated with the use of drug carriers. In recent years, much research has been carried out by modifying the surface of a wide range of DDSs such as liposomes and nanoparticles or conjugating different therapeutic agents with transferrin to achieve targeted delivery to the brain and thereby treat CNS diseases. The details of such delivery systems have been discussed in the following sections. For all systems, however, there are some limitations when employing transferrin as a targeting ligand. Transferrin binding can be limited by the endogenous concentrations of transferrin in the blood as transferrin receptors are always saturated by endogenous transferrin in circulation. Furthermore, using transferrin to bind transferrin receptors affects the cellular uptake of iron by the brain [71,72]. To overcome these limitations, alternatively, antibodies that bind to transferrin receptor via other sites different from transferrin have been discovered and are being investigated.

OX26 is an anti-transferrin receptor monoclonal antibody (MAb) that is recognized by transferrin receptor at extracellular domain without affecting transferrin binding to the receptor [73]. Similar to transferrin ligand, OX26 can be conjugated to various drug carriers or therapeutics to penetrate the BBB. Radio-iodinated Aβ, ^125^I-Aβ_40_ can be conjugated to OX26 by streptavidin-biotin linker to enhance its transportation through the BBB after intravenous injection for neuroimaging the Aβ in patients with AD. In this study, the transport of ^125^I-Aβ_40_ through the BBB was determined by capillary depletion [74]. When conjugating BDNF with PEG chains, the biological activity of BDNF is not changed and the distribution of BDNF in the spinal cord is increased. PEG-BDNF can be coupled with OX26 via streptavidin-biotin linker and penetrate the BBB via receptor-mediated transcytosis. In one study, the brain uptake of the BDNF-PEG-OX26/SA conjugate was increased to 0.15% of injected dose in each gram of SD rat brain after capillary depletion [75]. Peptide nucleic acids (PNAs) are therapeutics that are not able to cross the BBB. By conjugating PNA with OX26 via streptavidin-biotin linker, the brain uptake of PNA-OX26/SA was at least 28-fold greater than that of unconjugated PNA. And the author suggested the intracerebral concentrations of the drug were potentially high enough to be therapeutic significance for treating AD and cerebral AIDS [76]. However, it was not classified that whether the investigation was made with capillary deleted fraction of brain. Therefore, whether these molecules actually reached the brain parenchyma cannot be confirmed [77]. Similar to OX26, MAb 8D3 and RI7217 can also bind to transferrin receptors and cross the BBB. OX26 is anti-rat transferrin receptor MAb while 8D3 and RI7217 are anti-mouse transferrin receptor MAbs. A study was performed to evaluate the penetrability of these three MAbs in mice. Brain uptake of 8D3, RI7217 and OX26 were 3.1%, 1.6% and 0.06% respectively after intravenous injection, data were also demonstrated by capillary depletion analysis. Results indicated the brain targeting effects of MAb should be species-specific. Experimental results happened in mice or rat models may not happen in humans [78]. Therefore, appropriate choice of MAbs in designing the DDSs is significant for brain targeting. In order to overcome the species cross-reactivity problem, MAbs can also be modified by biochemical engineering techniques. One example has been provided in the following section.

#### 4.4.2. Insulin Receptor

Insulin receptors can be found on the surface of vascular endothelial cells and other brain cells [79]. Insulin is transported to the brain through receptor-mediated transcytosis. In the brain, glucose metabolism is regulated by insulin, insulin-like growth factors and their receptors. It has been reported that the concentrations of insulin and insulin receptors in the brain are correlated and decrease with aging. Sporadic AD is AD not exhibiting autosomal-dominant inheritance. In sporadic AD patients, brain insulin receptor density was decreased when compared to middle-aged controls but increased in comparison to age-matched controls. The insulin-immunoreactivity of sporadic AD patients was stronger than control groups [80]. The level of insulin in brain would affect the pathogenesis of AD. In details, insulin and Aβ are both degraded by insulin-degrading enzyme (IDE) with a priority for degrading insulin over Aβ. Therefore, a high level of insulin can slow the clearance of Aβ and result in a higher level of extracellular Aβ [81,82]. Furthermore, Aβ has been reported as a direct competitive inhibitor of insulin binding and action because they share a common sequence recognition motif. Both Aβ_1–40_ and Aβ_1–42_ can reduce the affinity of insulin for receptor. As insulin receptors play a significant role in glucose homoeostasis, impaired glucose metabolism would occur in AD patients [83]. Using endogenous insulin as a vector may not be an ideal choice for AD treatment. Two issues must be considered: serum half-life and insulin metabolism. The serum half-life of insulin is only 10 min and targeting the insulin receptors in brain may result in disturbance of insulin metabolism [53]. However, other MAbs have been utilized to bind insulin receptors for brain targeting. A mouse MAb against human insulin receptor (HIR), MAb83-14, is able to transport across the BBB in the Old-world primate, Rhesus monkey, with uptake of around 4% of the injected dose 3 h after injection [84]. Like other MAbs, MAb83-14 is also species-specific. It does not react with insulin receptors in New-world primates and rodents. In order to tackle the species-specific issue, fully humanized form of the 83–14 MAb have been produced through genetic engineering. The affinity of the humanized HIRMAb for the HIR extracellular domain was 27% lower than that of murine HIRMAb. In an in vitro model system of the human BBB, the humanized HIRMAb was bound to the HIR of isolated human brain capillaries. The humanized HIRMAb could also bind to the insulin receptors of Old-world primates such as Rhesus monkey. Therefore, the humanized HIRMAb can be evaluated in animal model and used to deliver therapeutics agents across the BBB in humans [85,86,87]. The humanized HIRMAb can be fused with an anti-Aβ MAb, scFv to treat AD. As a result, the conjugated product could bind to the human insulin receptor to enter the brain and target the Aβ fibril to disaggregate amyloid plaques. In the brain, the fusion protein was cleared by Fc receptor (FcRn), which is a receptor related to the efflux from brain to blood across the BBB [88]. The FcRn also regulates the efflux of human immunoglobulin (IgG). IgG is a MAb which can recognize both human transferrin and insulin receptors, with the ability to penetrate the BBB. IgG can also link with scFv to form a fusion protein. After intravenous administration in the adult Rhesus monkey, the fusion protein has been shown to penetrate the BBB but efflux from the brain to blood rapidly [89]. Although the HIRMAb are designed and engineered to bind with high affinity to a different receptor epitope from the endogenous insulin, there is still competition between endogenous ligands and HIRMAb-modified drug conjugates. It may change therapeutic efficacy, receptor activity and metabolism [90].

#### 4.4.3. Low Density Lipoprotein Receptor

Low density lipoprotein (LDL) receptors refer to a wide range of protein receptors that can recognize extracellular ligands for subsequent signaling, trafficking and scavenging in the brain. LDL receptor-related proteins receptor 1 (LRP1) and 2 (LRP2) are similar in structure to LDL receptor. The receptors are also expressed in brain capillary endothelial cells. Multiple ligands can bind to the receptors for receptor-mediated transcytosis; thus, they have been employed for targeted drug delivery to the brain [91,92,93]. The LRP is highly related to the pathology of AD. LRP1 is the receptor for ApoE, alpha2-macroglobulin (α2M) and transforming growth factor-beta (TGF-β) while LRP-2 is the receptor for apolipoprotein J (ApoJ). It was found that LRP1 can also bind with APP [94]. Besides ApoE and APP, α2M is also risk factors to AD. α2M is an abundant plasma protein having at least five binding sites including the receptor binding site and Aβ binding site so that it can bind to Aβ peptide tightly and also regulate the degradation and clearance of Aβ via endocytosis through LRP [95,96]. It has been reported that two polymorphisms in the α2M gene, A2M, are genetic risks for AD [97]. ApoE, α2M and LRP are inseparable in the dysfunction of Aβ clearance in AD. Therefore, therapy targeting the LRP-mediated pathway is being developed as a strategy for treating AD.

Lactoferrin (Lf) is a cationic iron-binding protein belonging to the transferrin family. It shows multifunctional properties including anticarcinogenic and anti-inflammatory activities [98]. Lf can bind to multiple receptors including LDL receptor-related protein receptor. It can be transported to the brain via LRP-mediated transcytosis [99,100]. It has been reported that the expression of lactoferrin is greatly upregulated in both neurons and glia in AD; therefore, it shows potential in brain targeting [101]. Another targeting ligand, melanotransferrin or melanoma tumor antigen p97, shares sequence similarity and iron-binding properties with members of the transferrin family such as lactoferrin. It also undergoes transcytosis via LRP receptor. Increase in melanotransferrin has been described in patients with AD [102]. After 1-h intravenous injection of melanotransferrin into mice, about 0.1% of the injected dose was found to have been delivered in the brains of the mice. In a bovine study, melanotransferrin transcytosis across bovine brain capillary endothelial cell monolayers was at least 14-fold higher than that of lactotransferrin. And the high transcytosis of melanotransferrin was not related to changes in the brain capillary endothelial cell monolayer integrity [103]. In another study, using ardimycin conjugated with melanotransferrin, the amount of P97-adrimycin transferred to the brain was found to be 6 to 8-fold greater as compared to lactoferrin [104].

There are still other ligands that can bind LRP to undergo receptor-mediated transcytosis. One example showed is receptor-associated protein (RAP). RAP is an endoplasmic reticulum (ER) resident chaperone required for the maturation of several members of the LDL receptor family. By studying the pharmacokinetics of [^125^I]-RAP transport into the brain in intact mice, it was found that 70% of [^125^I]-RAP was localized in the parenchyma rather than in the vasculature of the brain. And the [^125^I]-RAP was relatively stable in blood for 30 min. In situ brain perfusion in blood-free buffer showed that transport of [^125^I]-RAP across the BBB is a saturable process. However, the clearance of RAP by kidney and liver was rapid which may be a limitation for its application in drug delivery [105]. Despite promising results were shown in pre-clinical models for all mentioned receptor-mediated transcytosis, success in clinical trial has yet to be demonstrated.

## 5. Role of the Blood-Brain Barrier in Drug Delivery

The structure and the functions of the BBB are known much better than before. And some of the BBB dysfunctions in AD have also been summarized (Table 1). On the one hand, the BBB serves as a physical barrier that can protect the CNS from exogenous substances. On the other hand, the BBB serves as a transport system, delivering chemicals to the CNS. In the context of medicine, the BBB can be regarded as a target for DDSs, using various types of receptors expressed on the endothelial cells to transport therapeutics across the BBB. Strategies for delivering molecules into the brain can be classified as invasive and non-invasive. The invasive method is to directly administrate the drugs into brain. Approaches include temporarily opening the tight junctions by high osmolar solutions, injecting drugs intracerebrally or guiding drug injection via catheter [106,107]. However, invasive methods are inherently dangerous. There is risk of infection, potential damage to the brain tissue and possible uncontrolled drug distribution after administration. Therefore, employing non-invasive methods are generally preferred. In non-invasive approaches, the therapeutics can be administrated through intravenous injection or intranasal administration. In the nasal pathway, the drugs are able to enter the brain once penetrating the nasal epithelium [107]. The drugs will be delivered along olfactory and trigeminal neural pathways via extracellular route, which is the shortest path for molecules to reach the CNS. This approach does not require the drug to bind to any receptor for transcytosis. Nevertheless, the administration requires substances with high solubility as each dose is limited to about 20 to 30 μL. Both pH and salinity of the therapeutic formulation also influence the effectiveness of delivery. In extreme conditions, respiratory system may be damaged. Moreover, intranasal administration also requires skillful operations because improper injection may deliver the drug to lung or stomach [108]. As a result, intravenous administration is normally a common choice. Another non-invasive strategy for brain delivery is the use of focused ultrasound (FUS) to open the BBB transiently. With intravenous administration of microbubbles before sonication, BBB can be disrupted by low-frequency ultrasound waves. This is a promising strategy to achieve focal delivery of therapeutics including antibodies, nanoparticles and chemotherapies into the brain. And the BBB opening process is totally reversible [109]. It was reported that the magnetic resonance-guided focused ultrasound was applied to five patients with AD. Opening and restoration of the BBB was confirmed. The results were promising. However, the sample size in the study was small and the efficacy to treat AD was not studied [110]. More clinical data must be collected to verify the application of FUS in human.

In AD therapy, most of the compounds showing therapeutic efficacy are mostly lipophilic molecules with low water solubility. These molecules tend to be substrates for P-glycoprotein (ABCB1) and/or breast cancer resistance protein (BCRP/ABCG2), which means that uptake through the BBB is restricted by the P-glycoprotein and BCRP efflux [8,111]. In order to increase the uptake of drug molecules by the brain, different approaches can be considered. First, the structure of therapeutic molecules can be modified to adjust the physical and chemical properties. Synthesis of drug analogues is full of uncertainty because the activity site responsible for therapeutic effects and the properties of different functional groups have to be well studied. The synthesis usually requires several steps and sample loss is accumulated in each step. Conjugating the drug molecules with various cell penetrating peptides or antibodies is another strategy. This method can make use of the adsorptive-mediated transcytosis and receptor-mediated transcytosis pathways and facilitate the drug to cross the BBB (Table 2). Details of these pathways have already been discussed in previous section. Although ligand-conjugated drugs bring improvements for drug to delivery into the brain, problems such as the rate of drug dissociation from ligands and the ratio of drug/ligand and ligand/receptors still need to be solved. To improve the mentioned problems, nanotechnology-based DDSs are potential alternative to be considered.

## 6. Nanotechnology-Based Drug Delivery Systems for Penetrating the BBB to Treat AD

**Figure 2:**
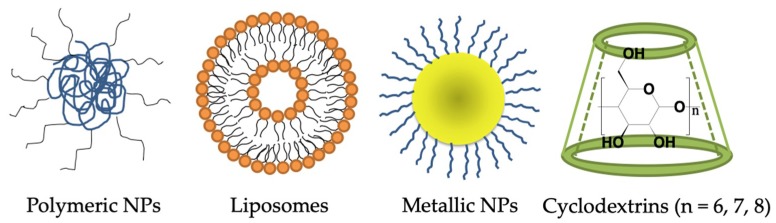
Different types of drug delivery systems.

For the purpose of delivering drugs across the BBB to treat AD, the use of DDS can be considered. Nano DDSs are composed of biodegradable materials such as natural or synthetic polymers, lipid and inorganic metal. The payload can be either entrapped in the matrix or attached to the surface of the DDS. By engineering the sizes, architectures and surface properties of the DDS, the effectiveness of delivering drugs to target sites can be maximized. DDS shows attractive benefits regarding drug targeting, delivery and release. After understanding the microenvironment of CNS and the target cell-surface receptors expressing in the brain of AD patients, a wide range of nanocarriers can be designed and employed for targeted drug delivery. In the following sections, nano DDSs (Figure 2) for crossing the BBB to treat AD have been divided into four categories and discussed [10,11,12,112].

### 6.1. Polymeric Nanoparticles

The size of nanoparticles (NPs) is within the range of 1 to 100 nm [113]. By adjusting the drug-to-polymer ratio and coating the surface of nanoparticles with surfactant, cell penetrating peptides or antibodies, properties of polymeric nanoparticles such as size, zeta potential, blood circulation time, drug releasing rate and targeting sites can be controlled. Biodegradable polymeric nanoparticles with appropriate surface modification that can deliver drugs for diagnostic and therapeutic applications in AD have already been reported (Table 3). These polymeric nanoparticles are usually composed of biodegradable or biocompatible polymers such as poly(lactic-co-glycolic acid) (PLGA), polylactic acid (PLA) and poly (butyl cyanoacrylate) (PBCA). PLGA are FDA-approved biodegradable and biocompatible copolymers of polylactic acid and polyglycolic acid (PGA). These copolymers degrade by hydrolysis in the body to generate monomeric components and they are removed from body through different natural mechanisms [114].

In recent research, phytol-loaded PLGA NP was prepared as a modulator of Aβ peptide aggregation in AD. The usage of phytol is limited due to its poor solubility in biological fluids. With drug-to-polymer ratio 1:4, drug-loaded polymeric NPs were successfully prepared with 92% encapsulation efficiency. These drug-loaded NPs sustained released of the drug for up to 120 h. The antioxidant efficacy, anti-aggregation and disaggregation of Aβ_25–35_ property, survival and protective effect against Aβ_25–35_ induced toxicity in Neuro2a cells of free phytol and phytol-loaded PLGA NPs were evaluated in vitro. The efficacy of the free drug or the drug-loaded PLGA were slightly improved when compared to the standard drug donepezil. This promising result indicates that the phytol-PLGA NPs might be applied as a novel therapeutic formulation for AD treatment. However, there was no in vivo data showing whether the phytol-PLGA NPs were able to penetrate the BBB to enter the brain [115]. Similar research was carried out by preparing galantine-loaded polymeric nanoparticles. Galantamine (GAL) has been approved for the treatment of mild to moderate AD. Loading the drug with PLGA and polysorbate 80 as the surfactant, GAL-loaded PLGA nanoparticles were obtained. The release of GAL from the nanoparticles was sustained as compared to the micellar and aqueous solutions. The cytotoxicity of the GAL-PLGA NPs was tested in two cell lines: HeLa cells and SH-SY5Y cells. Toxicity was only found in the SH-SY5Y cells at 3 mg/mL. For AchE inhibition, pharmacological activity of GAL maintained around 80% when encapsulated in nanoparticles. No in vivo data was provided in this study [116]. The above data indicates that encapsulating therapeutics with PGLA could modify the kinetics performance of the drug molecules. And the PLGA NPs may serve as a potential carrier for delivering drugs to the brain.

Various studies have reported that conjugation of peptides with drug-loaded PLGA NPs could increase the cellular uptake of NPs. Curcumin-encapsulated PLGA nanoparticles were first synthesized followed by conjugation with Tet-1 peptide through EDC-NHS coupling. TeT-1 attachment did not destroy the antioxidant activity of curcumin and the anti-amyloid activity of curcumin was not changed after encapsulation but with a slower inhibition rate as compared to naked nanoparticles [117]. The neuronal targeting efficiency of NPs in GI-1 glioma cells increased significantly when they were conjugated with Tet-1 peptide. The results suggest the potential of curcumin-PLGA NPs in treating AD and the effect of Tet-1 peptide in neuronal targeting of the nanoparticles [117]. Curcumin was also encapsulated with a formulation based on PLGA and a stabilizer polyethylene glycol, PEG-5000. Bioavailability studies of curcumin-PLGA NPs showed that the serum levels of curcumin were almost twice as high in NPs group as compared to free curcumin group. In addition, the half-life of curcumin-PLGA NPs was also substantially longer than that of free curcumin [118]. More research has already been carried out in a mice model. A simple curcumin-loaded PLGA NP was prepared with encapsulation efficiency of about 77%. The curcumin-PLGA-NPs were internalized into neural stem cells and neurospheres after 3 h of treatment in vitro and crossed the BBB of rats after intraperitoneal administration in vivo. Curcumin was sustained and slowly released from the NPs. The levels of curcumin in the brain of curcumin-PLGA-NP-treated groups were increased 2.1- to 2.8-fold as compared to similar doses of bulk curcumin. Improved cognitive deficits in Aβ-treated rats were observed in curcumin-PLGA-NPs treatment group as compared to control and bulk curcumin-treated groups. Curcumin-PLGA-NPs still showed effectiveness even at a dose of 0.5 mg after intrahippocampal stereotaxic injection, while bulk curcumin showed no significant enhancement of neurogenesis at the same dose. Curcumin-PLGA-NPs also inhibited Aβ-induced neurodegeneration in the hippocampus and enhanced learning and memory abilities in AD rat model by activation of the Wnt/β-catenin signaling. The results proved that curcumin nanoparticles may be a novel therapy for AD [119]. Recently, a brain targeting CRT peptide-conjugated PLGA NP loaded with Aβ generation inhibitor S1 (PQVGHL peptide) and curcumin was developed. CRT (sequence: CRTIGPSVC) is a cyclic iron-mimic peptide that targets transferrin receptor. CRT peptide increased the permeation of PLGA NPs across in vitro BBB model built up by microvascular bEnd3 cells. In a biodistribution study, more CRT-PLGA NPs accumulated in the mouse brain when compared to PLGA NPs without CRT conjugation. The CRT-PLGA NPs loaded with S1 and curcumin were still observed in the brains of the sacrificed mice after 24 h of intravenous administration. The drug-loaded CRT-PLGA NPs did not possess significant cytotoxic effect on SH-SY5Y cells, microglial BV2 cells and bEnd3 cells. The polymeric system significantly decreased Aβ burden, gliosis and inflammation in the brains of AD mice and increased spatial memory and recognition [120]. There are more examples of using polymeric nanoparticles for AD treatment. Dexibuprofen-loaded pegylated PLGA nanospheres were designed to increase dexibuprofen brain delivery and reduce systemic side effects. The drug-loaded nanospheres were not toxic to brain endothelial cells and astrocytes and did not cause the BBB disruption. The permeation coefficient of dexibuprofen was increased and the nanospheres were able to cross the BBB of the mice in vivo after oral gavage administration. The concentration of nanospheres in the brain was detected at a concentration of about 0.37 mg/mL of nanospheres per gram tissue. Behavioral tests performed in an APPswe/PS1dE9 AD mice model demonstrated that dexibuprofen-loaded nanospheres showed better efficiency than the free drug in reducing memory impairment. Such results showed that dexibuprofen pegylated PLGA nano systems could be suitable for AD treatment [121]. There are other studies using polymers other than PLGA. PEGylated nanoparticles of poly[hexadecyl cyanoacrylate-co-methoxypoly(ethylene glycol) cyanoacrylate] (P(HDCA-co-MePEGCA)) was reported to exhibit affinity towards the Aβ_1–42_ peptide. Therefore, poly[(hexadecyl cyanoacrylate-co-rhodamine B cyanoacrylate-co-methoxypoly(ethylene glycol cyanoacrylate)] (P(HDCA-co-RCA-co-MePEGCA)) and poly[methoxypoly(ethylene glycol) cyanoacrylate-co-Biotin-poly(ethylene glycol) cyanoacrylate-co-hexadecyl cyanoacrylate] (MePEGCA-co-Bio-PEGCA-co-HDCA) copolymers were employed to prepare non-functionalized NPs. The NPs were then decorated anti-Aβ_1–42_ MAb. In biodistribution study of anti-Aβ_1–42_-NPs in mice model; approximately 0.5% of the NPs were detected in the brain after intravenous injection. Therapeutic efficacy of anti-Aβ_1–42_-NPs to reduce soluble forms of Aβ and rescue memory in AD mice was effective. The treatment did not induce any significant reduction of insoluble brain Aβ burden but did significantly reduce by 20% of the Triton-soluble Aβ_1–40_ and Aβ_1–42_ levels in the brain when compared to control group. Treatment of anti-Aβ_1–42_-NPs also reduced the amount of Aβ oligomers. After treatment with anti-Aβ_1–42_-NPs for three weeks, a significant increase of about 20% in Aβ levels in plasma was detected when compared to the mice treated with PBS, which implied that Aβ peptides were cleared from the brain into blood circulation [122].

### 6.2. Lipid-Based Nanocarriers—Liposome

Liposomes are sphere-shaped vesicles consisting of one or more phospholipid bilayers. They have an aqueous core that can be used to encapsulate hydrophilic molecules and they have a lipid bilayer that can trap lipophilic particles. Therefore, a wide range of moieties can be loaded onto liposomes. Generally, the particle size of liposomes is 30 nm to several um. Depending on the composition of the formulations, properties such as the size, zeta potential and rigidity of the liposomes can be controlled [123]. The surface of the liposomes can be modified for different purposes. PEGylation of liposomes can improve the pharmacokinetics and pharmacodynamics of the biomolecules [124]. Specific targeting actions of liposomes can be achieved by conjugating suitable targeting vectors. For drug delivery to the brain, the use of cationic liposomes facilitates crossing the BBB through adsorptive-mediated transcytosis [68]. By coupling liposomes with cell-penetrating peptides and antibodies, as mentioned in previous sections of this review, liposomes can also deliver therapeutic molecules across the BBB through receptor-mediated transcytosis. In recent years, many liposomal DDSs have been established to treat AD (Table 4).

Rivastigmine is one of the FDA-approved drugs to treat AD; it has a short plasma elimination half-life of about 1.5 h and its brain penetration is restricted by tight junctions. When formulations were administered orally and intraperitoneally in mice, by encapsulating rivastigmine in dipalmitoylphosphatidyl choline (DPPC)/cholesterol liposomes increased delivery of the drug into the brain as compared to drug administration without liposomes [125]. Peptides can also be loaded onto liposomes. H102 is a novel β-sheet breaker peptide with sequence HKQLPFFEED. Encapsulating H102 with egg phosphatidylcholine (EPC) and cholesterol, H102 peptide-loaded PEGylated liposomes was prepared. The stability of the peptide was enhanced. After intranasal administration, H102 could be delivered to the brain effectively and the AUC of H102 liposomes in the hippocampus was 2.92-fold larger than the peptide solution group. Liposomal H102 improved the spatial memory impairment, increased the activities of choline acetyltransferase (ChAT) and insulin degrading enzyme (IDE) and inhibit plaque deposition in an AD rat model better than the solution group. Furthermore, the formulation did not show toxicity on nasal mucosa [126]. More research has been conducted with targeting vectors-modified liposomes. A potential agent to treat AD, α-Mangostin (α-M), was delivered to the brain by transferrin-modified liposomes. The liposomes mainly consisted of 1,2-distearoyl-sn-glycero-3-phosphatidylcholine (DSPC) and cholesterol, with transferrin conjugated to the PEG chains on the surfaces of the vehicles. In this study, in vitro and in vivo experiments were carried out to evaluate the ability of the transferrin-modified liposomes to cross the BBB. An in vitro model of BBB using bEnd3 cells and astrocytes was established. The concentration of α-M of Tf-α-M-liposome was higher than that of free α-M and conventional α-M liposome. Integrated liposomes could still be observed by TEM after penetrating the in vitro barrier. The pharmacokinetic parameters indicated that Tf-α-M-liposome could improve the bioavailability of the α-M in the plasma when compared to free α-M. In addition, more α-M accumulated in the brain of the Tf-α-M liposome group. The results suggest that transferrin-modified liposomes can bind with the Tf receptors expressing on the surface of the BBB, thereby transporting the payload into the brain [127]. Lactoferrin-modified liposomes were also studied as a promising drug delivery cargo to treat AD. Procationic liposomes are liposomes that are negatively charged or electrically neutral at a physiological pH and change into cationic liposomes when they enter the brain. With the help of lactoferrin, compared to the conventional liposomes, the uptake of Lf-PCLs in in vitro BBB model and in mice brain was higher. The endocytosis of Lf-PCL crossing the BBB was involved in both receptor- and adsorptive-mediated transcytosis [99]. A neuron growth factor (NGF) -loaded liposomes with surface lactoferrin were developed. The kinetics of NGF release from the liposomes was studied. Lf-NGF-liposomes could release about 72% to 90% of NGF in 48 h. The toxicity and permeability were determined in human brain microvascular endothelial cells (HBMECs) and human astrocytes (HAs) cells in vitro [128]. Similar to previous results [99], lactoferrin accelerated drug delivery across the BBB and also increased the viability of neuron-like SK-N-MC cells. Moreover, Lf-NGF-liposomes also inhibited the degeneration of SK-N-MC cells with Aβ-induced neurotoxicity. The Lf-NGF-liposomes showed its potential in AD treatment. However, in vivo data should be obtained from further experiments [128]. Dual functionalized liposomes were also developed by grafting Lf-liposomes with RMP-7, a bradykinin analog with high selectivity in binding to the BBB. Quercetin was encapsulated in the RMP-7-Lf-liposome to traverse HBMECs and to treat SK-N-MC cells after an insult with Aβ fibrils. The toxicity and permeability of RMP-7-Lf-quercetin-liposome was also determined in HBMECs and HAs. The liposome crossed the BBB without inducing strong toxicity or damaging the tight junction. Results also suggested that the dual functionalized liposome might slightly enhance paracellular drug delivery. The liposome could also protect SK-N-MC cells from significant neurodegeneration caused by Aβ-induced neurotoxicity. It could also inhibit the expression of phosphorylated p38 and phosphorylated tau protein at serine 202 by SK-N-MC cells [129]. The RMP-7-Lf-liposome appears to be a promising drug carrier for future development. More in vivo data are needed.

Other targeting vectors have also been employed to develop liposomal-based nano DDSs for targeted delivery to brain. OX26 mono-decorated liposomes, RI7217 mono-decorated liposomes, ApoE3 mono-decorated liposomes, OX26-ApoE3 dual-functionalized liposomes and RI7217-ApoE3 dual-functionalized liposomes have been synthesized for BBB targeting. All of the liposomes were nontoxic to hCMEC/D3 cell monolayer. The uptake of the liposomes was significantly affected by the conjugated targeting vectors. The cellular uptake of dual-functionalized liposomes was nearly two folds higher as compared to the mono-functionalized liposomes. The uptake of dual-functionalized liposome in vitro showed an additive effect. However, in vivo data of mice model showed that OX26, RI7217 mono-decorated liposomes and dual-functionalized liposomes increased brain targeting while ApoE mono-decorated liposome did not when compared to non-targeted liposomes. The brain targeting of dual-functionalized liposomes did not show addictive effect. Results from in vivo study with ApoE3 did not comply with the data observed from in vitro experiments. Such results were explained by in vitro studies in the presence of serum proteins. The later result implies that the ApoE peptide was inactivated by serum proteins. This study could be great significance when considering strategies to design novel targeted DDSs. Different types of MAb or cell-penetrating peptides may not perform well in an in vivo environment, even they showed good results under in vitro studies [130]. Following are other successful examples of dual-functionalized liposomes. Phosphatidic acid (PA) and mApoE (sequence: CWG-LRKLRKRLLR) dual-modified liposomes were reported to show therapeutic effectiveness in transgenic mice model. The dual-functionalized liposomes hindered the formation and disaggregated of Aβ assemblies in vitro. Intraperitoneal administration of dual-functionalized liposomes to APP/presenilin 1 transgenic mice for 3 weeks decreased total brain-insoluble Aβ_1–42_ by 33% and the number and total area of plaques by 34% when compared to control group. Also, brain Aβ oligomers were reduced around 70.5%. Mouse impaired memory was ameliorated. Although the mApoE-PA-liposome could not eliminate the cause of Aβ overproduction, it could certainly slow the neurodegeneration process [131]. A multifunctional nanoliposome was developed by modifying its surface with a lipid-PEG-curcumin derivative and additionally decorating it with an anti-transferrin antibody. Curcumin and its derivatives were able to bind to the amyloid deposits in vivo. Therefore, lipid-PEG-curcumin derivative was designed to target amyloid deposits. The brain targeting capability of the multifunctional nanoliposome was investigated. It was proven that the presence of the lipid-PEG-curcumin derivative on the surface did not affect the binding of anti-transferrin antibody. A brain from AD patients was used to evaluate the effectiveness of the multifunctional nanoliposome. Results verified that the attachment of an antibody on the curcumin-liposome surface did not block deposit staining or prevent Aβ aggregation. In this case, the nanoliposome can take advantages of both the conjugated moieties and achieve targeted delivery to treat AD [132].

### 6.3. Metal-Based Nanoparticles

The use of metal-based nanoparticles to treat AD is relatively less common when compared to that of polymeric nanoparticles and lipid-based carriers (Table 5). Metal nanoparticles are between 1 and 200 nm in size and they show special physicochemical characteristics depending on their size and shape. There are different types of metallic NPs such as gold, silver, titania and so forth [133]. The effects of size, charge and shape of gold nanoparticles (AuNPs) on Aβ aggregation was studied. AuNPs of size 20, 50 and 80 nm with the same total surface area were used to study the effect of size. To obtain same total surface area, total surface area of 50-nm AuNPs (50 pM) were first calculated, then the amount of 20-nm and 80-nm AuNPs were adjusted. Results showed that larger AuNPs induced large and amorphous aggregates on the brain lipid bilayer while smaller AuNPs induced protofibrillar Aβ structures. Amine-modified AuNPs with positive charges and citrate-modified AuNPs with negative charges were synthesized to study the effect of charge. Positively charged AuNPs could attach to Aβ stronger than the AuNPs with negative charge. The stronger interactions between AuNPs and Aβ led to fewer β-sheets and more random coil structures. Gold nanorods (AuNRs) and gold nanocubes (AuNCs) were also synthesized to study the effect of shape on Aβ aggregation. Aβ was preferentially bound to the long axis of AuNRs and fewer fibrils were formed while all the facets of AuNCs interacted with Aβ to produce the fibril networks. The size, shape and surface charge of NPs can be controlled during synthesis. This study provides information for design a DDS using AuNPs [134]. It was reported that transferrin modified AuNPs and AuNRs could cross the BBB both in vitro and in vivo. With the use of near infrared light (NIR) irradiation under certain concentration, the BBB could be opened without significantly reducing cell viability and the gold nano-formulations could preferentially accumulate in the neurogenic regions of the brain in mice model. Results also offered direction to develop gold-based DDSs combining the use of transferrin peptides and laser activation to treat neurodegenerative diseases [135]. An AuNPs-based multifunctional Aβ Inhibitor against AD was developed. First, the surface of AuNPs was grafted using an Aβ Inhibitor, polyoxometalates with Wells–Dawson structure (POMD) and then conjugated with LPFFD peptide, which served as β-sheet blocker. Results showed synergistic effects in inhibiting Aβ aggregation, dissociating Aβ fibrils and decreasing Aβ-mediated peroxidase activity and Aβ-induced cytotoxicity in vitro. This DDS was able to cross the BBB in mice model when administered via tail vein injection [136].

### 6.4. Others—Cyclodextrins

Cyclodextrins (CDs) are cyclic oligosaccharides which have a cage-like supramolecular structure. The inner cavity of can hold lipophilic molecules and form water soluble guest/host complexes without forming covalent bonds. CD inclusion complexes can serve as DDSs. Solubility, bioavailability and stability of payloads will be increased after insertion into the CDs [137]. CDs can act as therapeutic agents to treat neurodegenerative diseases due to their ability of lipid extraction [138]. It was reported that CDs showed neuron protective effects by reducing Aβ production and enhancing clearance mechanisms both in a cell model and a transgenic mouse model of AD. Hydroxypropyl-β-CD (HP-β-CD) was employed for evaluation. It could reduce the levels of Aβ_1-42_ and membrane cholesterol in SwN2a cells. After treating transgenic mice in an AD mice model for 4 months by subcutaneous administration, spatial learning and memory of the mice were improved, Aβ plaque deposition was diminished and tau immunoreactive dystrophic neurites were reduced. It proved that HP-β-CD could reduce β cleavage of the APP protein and up-regulate the expression of genes involved in cholesterol transport and Aβ clearance [139]. CDs can also act as drug carriers to increase the permeability of lipophilic drugs into the brain parenchyma [140]. Co-incubation of various β-CDs with doxorubicin for BBB penetration was performed on an in vitro model. Rame-β-CD and Crysme-β-CD increased the transport of doxorubicin by factors of 2- and 3.7-fold, respectively. The increase was due to the cholesterol extraction property of CDs and thus led to a modulation of the P-gp activity [141]. A polymeric nanoparticle composed of β-CD and poly(β-amino ester) segments was developed for doxorubicin drug delivery across the BBB. Bovine and human brain microvascular endothelial cell monolayers were constructed as in vitro BBB models. Results showed that the delivery system did not affect the integrity of the in vitro BBB models and it had high permeability across the in vitro BBB models [142]. Various researches have showed the potential therapeutic use of CDs to deliver drugs across the BBB and treat AD (Table 5) [138,140]. It is hoped that encapsulating therapeutic molecules for AD treatment with CDs will yield additive effects and achieve promising results in treating neurodegenerative diseases.

## 7. Discussion and Future Perspective

Although the cause of AD is still not fully understood and it is hard to diagnose the disease at its early stage, more and more evidence supports the hypothesis that Aβ peptides and tau proteins are at the core of AD pathogenesis. According to the Aβ hypothesis, pathogenesis of AD is due to imbalance in the production and clearance of Aβ peptides in the brain. Based on this rationale, the most direct strategies are to reduce the Aβ peptides production and to clear the Aβ peptides out of the brain. Therefore, Aβ peptides and the pathway of generating Aβ peptides can be targets for development of novel therapeutic candidates. Targeting tau protein should also show positive results in treating AD. Targeting Aβ peptides and tau proteins simultaneously may deliver the strongest therapeutic effect. The potential for inhibiting Aβ and tau protein production or aggregation is perhaps an important criterion when selecting novel drugs to treat AD. Meanwhile, the properties of the BBB also needed to be considered.

**Figure 3:**
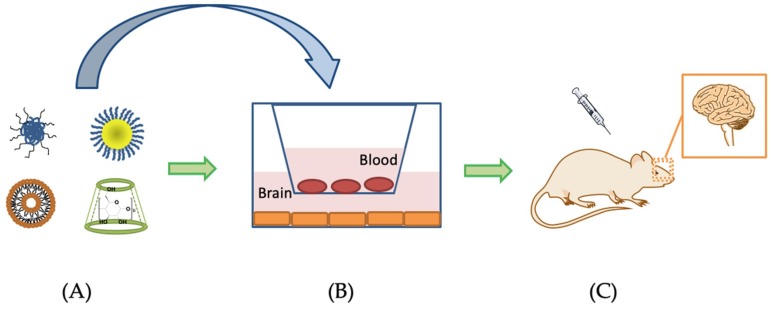
Scheme of designing DDSs to cross the BBB and treat AD. (**A**) Formulation development; (**B**) In vitro BBB model to predict the permeability of the DDSs; (**C**) Test the therapeutic efficacy of formulation in animal models.

Based on the mechanism of the BBB permeation, various cell penetrating peptides or antibodies can make use of the adsorptive-mediated transcytosis and receptor-mediated transcytosis pathways and facilitate the transport of drugs across the BBB. By conjugating the targeting vectors onto the surface of drug carriers, properties of the DDSs can be modified. However, special attention should be paid to the effectiveness and quality control of the DDSs. In some cases, in vitro results and in vivo results may show contradictions. This is due to the variations between the in vitro model and in vivo environment [130]. Some of the in vitro models are leaky and not polarized which may lead to wrong results [143]. Up to now, there is no perfect cell culture model to simulate the BBB. The properties of the BBB are changed during AD. Using different cell models may be possible to study the specific disease state as BBB permeability and drug permeation may vary in different pathologies (Figure 3). Therefore, choosing appropriate in vitro models may help to improve the simulation of in vivo environment [144]. In future, DDS will play a more important role in brain targeting due to its attractive advantages. Multi-functionalized DDS appears to be more effective than mono functionalized DDS. Combining different DDS can also be considered. For example, a drug-in-cyclodextrin-in-liposome system can take advantage of both biomaterials and thereby possibly achieve greater therapeutic efficacy [145].

## 8. Conclusions

The biology of the BBB and the BBB permeation mechanisms are vital issues to be considered when designing DDSs. Better knowledge of the pathophysiology of AD may provide more directions to break through the failures of current treatments for AD. Various DDSs show attractive properties for targeted drug delivery to the brain. Some of the latest applications of DDSs have been discussed in this review article. By optimizing the composition of the carriers and choosing appropriate targeting vectors, multi-functionalized DDS can be obtained, which can play a vital role in AD therapy. Developing DDSs with high therapeutic efficacy and minimum side effects may require the cooperation of experts from different fields. At this moment, the use of nanotechnology-based DDSs appears to be a promising direction for AD therapy and this strategy will become more developed in future as the pathophysiology of AD will be elucidated.

## Figures and Tables

**Table 1:** Features of blood-brain barrier dysfunction in AD.

Component	Features in AD	Ref.
Capillaries	Total length is shorter.	[36]
GLUT1	Downregulate, result in reduction of Aβ clearance	[51]
Transferrin receptor	Number of receptors in hippocampus are less than normal.	[70]
Insulin receptor	Brain insulin receptor density decreases with aging.	[80]
Lactoferrin	Expression is upregulated.	[101]
Melanotransferrin		[102]

**Table 2:** Summary of different brain targeting vectors.

Targeting Vectors	Pathway	Features	Ref.
Syn-B vectors	Adsorptive-mediated transcytosis	Cross the BBB without compromising the BBB integrity	[55,56,57]
TAT-derived peptides		Penetrating ability is related to the arginine residues in TAT. Non-specific transduction, induce Aβ deposition, tau phosphorylation and subsequent neuronal death in AD development	[60,61,62,63,64,65]
Polyarginines		Highly hydrophilic and cationic nature is responsible for charge repulsion which makes endocytosis possible.	[66]
Transferrin	Receptor-mediated transcytosis (Transferrin receptor)	Compete with endogenous transferrin in the blood, affect cellular uptake of iron by the brain	[71,72]
OX 26		Bind at extracellular domain without affecting transferrin binding, brain targeting effect is species-specific.	[73,74,75,76,78]
MAb 8D3			
RI7217			
Insulin	Receptor-mediated transcytosis (Insulin receptor)	Affect the clearance of Aβ and result in higher level of extracellular Aβ, short serum half-life, disturb insulin metabolism	[53,80,81,82,83]
MAb83-14		Species-specific. Only transport across the BBB in Old-World primates	[84]
HIRMAb		Can be evaluated in animal model and humans	[85,86,87]
IgG		Recognize both human transferrin and insulin receptors with ability to penetrate the BBB	[89]
ApoE	Receptor-mediated transcytosis (Low density lipoprotein receptor)	APOE4 allele is a genetic risk factor for late-onset AD, ApoE can regulate the integrity of tight junctions	[31,32]
Lactoferrin		Expression is greatly upregulated in both neurons and glia in AD.	[98,99,100,101]
Melanotransferrin		Stronger BBB-penetrating ability than lactotransferrin in bovine, not change integrity of the brain capillary endothelial cell monolayer	[102,103]

**Table 3:** Examples of polymeric nanoparticles used to treat AD.

Encapsulated Agents	Carrier Composition	Targeting Vectors	Elevated Model	Therapeutic Effects	Ref.
Phytol	PLGA, PVA	--	Neuro2a cells, without in vivo data	Sustained Release, show anti-amyloid activity, show neuron protective effect	[115]
Galantamine	Polysorbate 80, PLGA	--	HeLa cells, SH-SY5Y cells, without in vivo data	Sustained release, show AchE inhibition ability	[116]
Curcumin	PLGA, PVA	Tet-1 peptide	GI-1 glioma cells	Show anti-amyloid activity, show neuronal targeting effect	[117]
Curcumin	PLGA-PEG-5000	--	Mice	Increase drug serum level, longer half-life	[118]
Curcumin	PLGA, PVA	--	Neural stem cells, neurospheres, rats	Internalized by cells in vitro, cross the BBB in vivo, improve memory and cognitive ability, inhibit Aβ-induced neurodegeneration	[119]
Curcumin	PLGA-PEG-3400	CRT peptide and S1 inhibitor	bEnd3 cells, SH-SY5Y cells, BV2 cells, mice	Nontoxic to neuron cells, decrease Aβ burden, gliosis and inflammation in vivo, improve spatial memory and recognition	[120]
Dexibuprofen	PLGA-PEG, PVA	--	PC12 cells, bEnd3 cells, glial cells, APPswe/PS1dE9 mice	Nontoxic to cells in vitro, increase the BBB permeation coefficient, reduce memory impairment	[121]
--	P(HDCA-co-RCA-co-MePEGCA), MePEGCA-co-Bio-PEGCA-co-HDCA	Anti-Aβ_1–42_ MAb	Tg2576 mice	Reduce triton-soluble Aβ peptides and oligomers levels in the brain	[122]

“--”: means not applicable.

**Table 4:** Examples of liposomes used to treat AD.

Encapsulated Agents	Carrier Composition	Targeting Vectors	Elevated Model	Therapeutic Effects	Ref.
Rivastigmine	Cholesterol, DPPC, Methyl cellulose, dimethyl-β-CD, sodium taurocholate	--	Balb-C type mice	Increase amount of drug delivered into the brain	[125]
H102 peptide	EPC, DSPE-PEG2000 and cholesterol	--	SD rat	Enhance peptide stability, increase amount of peptide delivered into the brain, improve spatial memory impairment, increase activities of ChAT and IDE	[126]
α-Mangostin	DSPC, cholesterol, DSPE-PEG2000, DSPE-PEG2000-COOH	Transferrin	bEnd3 cells, astrocytes, SD rat	Penetrate in vitro BBB model without destroying the structure of liposomes, improve bioavailability of drug and increase amount of drug in brain	[127]
--	CHETA, DDAB, DOPE, PC	Lactoferrin	BCE cells, astrocytes, Kunming Mice	Enhance the uptake of Lf-procationic liposomes	[99]
NGF	DPPC, DSPE-PEG2000, DSPE-PEG2000-CA	Lactoferrin	HBME cells, human astrocytes, SK-N-MC cells, without in vivo data	Accelerate drug delivery across the BBB model, prevent Aβ-induced neurotoxicity	[128]
Quercetin	DPPC, cardiolipin, DSPE-PEG2000-CA, SPC, stearylamine, cholesterol	Lactoferrin, RMP-7	HBME cells, SK-N-MC cells, human astrocytes, without in vivo data	Slightly enhance paracellular drug delivery, protect neurodegeneration caused by Aβ-induced neurotoxicity	[129]
--	DSPC, DSPE-PEG2000, DSPE-PEG-Mal, DSPE-PEG2000-biotin	OX 26/RI7217/ApoE3/OX26 + ApoE3/RI7217 + ApoE3	hCMEC/D3 cells, FVB Mice	Cellular uptake of dual functionalized-liposomes was nearly twice as compared to mono-functionalized liposomes. In vivo results did not comply with in vitro results as ApoE peptide was inactivated by serum proteins.	[130]
--	Sphingomyelin and cholesterol	Phosphatidic acid, mApoE	APP/presenilin 1 mice	Decrease total Aβ fibrils and oligomers in brain, slow neurodegeneration	[131]
--	DSPC, DSPE-PEG2000 and cholesterol	Lipid-PEG-curcumin derivative, OX26, RI1227	Brain from AD patient	Able to bind amyloid deposits	[132]

“--”: means not applicable.

**Table 5:** Examples of Metallic nanoparticles and cyclodextrin-based delivery system to treat AD.

Encapsulated Agents	Carrier Composition	Targeting Vectors	Elevated Model	Therapeutic Effects	Ref.
--	AuNPs	--	--	Large AuNPs induce amorphous aggregates on the brain lipid bilayer. Smaller AuNPs induce protofibrillar Aβ structures.	[134]
--	Amine-modified AuNPs, Citrate-modified AuNPs	--	--	Positively charged AuNPs attached to Aβ more tightly.	[134]
--	AuNRs, AuNCs	--	--	Aβ was preferentially bound to the long axis of AuNRs and fewer fibrils were formed. All the facets of AuNCs interacted with Aβ to produce the fibril networks.	[134]
--	AuNPs, AuNRs	Transferrin	CD34^+^-derived ECs with bovine pericytes, C57BL/6 inbred strain mice	Cross the BBB both in vitro and vivo. With the use of NIR, irradiation, formulations could preferentially accumulate in the neurogenic niches.	[135]
--	AuNPs	POMD, LPFFD peptide	PC12 cells, S4880202 normal mice	Synergistic effects in inhibiting Aβ activity and Aβ-induced cytotoxicity in vitro, penetrate the BBB in vivo	[136]
HP-β-CD	HP-β-CD	--	SwN2a cells, Tg19959 mice	Reduce the levels of Aβ42 and membrane cholesterol in vitro, improve spatial learning and memory, reduce Aβ plaque deposition and tau immunoreactive dystrophic neurites in vivo	[139]
Doxorubicin	Rame-β-CD or crysme- β-CD	--	BCE cells	Increase the transport of doxorubicin, modulate P-gp activity	[141]
Doxorubicin	β-CD, poly(β-amino ester)	--	BME cells	High permeability across the in vitro BBB models	[142]

“--”: means not applicable.
